# Dietary omega-3 polyunsaturated fatty acid and alpha-linolenic acid are associated with physical capacity measure but not muscle mass in older women 65–72 years

**DOI:** 10.1007/s00394-021-02773-z

**Published:** 2021-12-16

**Authors:** Masoud Isanejad, Behnam Tajik, Anne McArdle, Marjo Tuppurainen, Joonas Sirola, Heikki Kröger, Toni Rikkonen, Arja Erkkilä

**Affiliations:** 1 Department of Musculoskeletal and Ageing Sciences, Institute of Life Course and Medical Sciences, University of Liverpool, Liverpool, L6 8TX England, UK; 2 Institute of Public Health and Clinical Nutrition, University of Eastern Finland, Kuopio Campus, Kuopio, Finland; 3 Kuopio Musculoskeletal Research Unit, University of Eastern Finland, Kuopio Campus, Kuopio, Finland; 4 Department of Obstetrics and Gynaecology, Kuopio, University Hospital, Kuopio, Finland

**Keywords:** n-3 PUFA, Alpha-linolenic acid, Muscle mass, Physical function

## Abstract

**Purpose:**

The aim was to investigate the cross-sectional association of dietary omega-3 polyunsaturated fatty acids PUFA (alpha-linolenic acid (ALA), eicosapentaenoic acid (EPA), and docosahexaenoic acid (DHA)) intake with multiple physical functions, muscle mass and fat mass in older women.

**Method:**

Study subjects were 554 women from the Osteoporosis Risk Factor and Prevention Fracture Prevention Study, with dietary intake assessed with 3-day food record. Body composition was measured by dual-energy X-ray absorptiometry. Physical function measures included walking speed 10 m, chair rises, one leg stance, knee extension, handgrip strength and squat. Short physical performance battery (SPPB) score was defined based on the European working group on sarcopenia criteria.

**Results:**

The multivariable adjusted models showed statistically significant associations for dietary ALA with higher SPPB (*β* = 0.118, *P* = 0.024), knee extension force at baseline (*β* = 0.075, *P* = 0.037) and lower fat mass (*β* = − 0.081, *P* = 0.034), as well as longer one-leg stance (*β* = 0.119, *P* = 0.010), higher walking speed (*β* = 0.113, *P* = 0.047), and ability to squat to the ground (*β* = 0.110, *P* = 0.027) at baseline. Total dietary omega-3 PUFA was associated with better SPPB (*β* = 0.108, *P* = 0.039), one-leg stance (*β* = 0.102, *P* = 0.041) and ability to squat (*β* = 0.110, *P* = 0.028), and with walking speed (*β* = 0.110, *P* = 0.028). However, associations for dietary EPA and DHA with physical function and body composition were not significant.

**Conclusion:**

Dietary omega-3 and ALA, but not EPA and DHA, were positively associated with muscle strength and function in older women. The intake of omega-3 and its subtypes was not associated with muscle mass. Longitudinal studies are needed to show whether omega-3 intake may be important for muscle function in older women.

## Introduction

Ageing is associated with progressive physical function loss, changes in body composition by increase in fat mass and loss of muscle mass. These can lead to independence loss, frailty and consequently failing to carry on with activities of daily living, mental and cognitive decline [1]. Variety of assessment tools are currently available to asses physical function decline in older people, e.g. walking speed, hand grip strength, chair rises, knee extension force which are predictors of physical capacity, falls and hospitalization [2, 3], morbidity, and mortality [4, 5]. These physical function assessments are also highlighted as significant components of sarcopenia [6] and Fried frailty definition [7]. Similarly, muscle mass loss in older adults has been suggested as a clinical predictor of these adverse health outcomes [8, 9].

Investigating the role of diet and nutrients in preventing the onset of sarcopenia, deterioration of physical function and muscle mass loss are of high public health interest, mainly owing to the possibility of altering these factors via related interventions [10, 11]. Among the components of a healthy diet, omega-3 PUFA are under focus in ageing research. The three main omega-3 PUFA are alpha-linolenic acid (ALA), eicosapentaenoic acid (EPA), and docosahexaenoic acid (DHA). Humans are unable to synthesize ALA, and as a result, it is essential to the diet. ALA is found mainly in plant oils such as flaxseed, soybean, and canola oils, whereas DHA and EPA are found in fish and other seafood [12]. These fatty acids and their food sources such as fish are embedded in public health recommendations for their beneficial health effects including anti-inflammatory properties [13], and they are suggested to have a role with multiple health outcomes [14] including muscle health [15, 16]. Noteworthy that WHO consortium on healthy ageing 2020 suggested that more research into role of omega-3 PUFA and healthy ageing are needed [17].

Muscle mass is maintained by a balance between muscle protein synthesis and breakdown. It has been proposed that n-3 PUFAs may be linked to muscle synthesis by enhancing the muscle protein synthesis or mTOR pathway. Finding from experimental studies, suggest that total EPA and DHA contributed to muscle protein synthesis and muscle anabolic stimulation [18]. In addition, in vivo studies have reported the changes in skeletal muscle phospholipid composition which can increase the amino acid uptake by these cells, triggering muscle anabolism [18, 19].

An important cause of loss of muscle mass and strength with ageing is low grade inflammation. A systematic review and meta-analysis by Tuttle et al., indicated that higher levels of circulating inflammatory cytokines are associated with lower skeletal muscle strength and muscle mass. Hence, a plausible pathway of dietary omega-3 PUFAs in prevention loss of muscle mass and strength can be reducing low grade inflammation [20–22] in older people.

Observational studies provided evidence on the positive association between plasma phospholipid PUFAs with knee extension and muscle mass [23], total omega-3, DHA, EPA and ALA intakes were positively associated with peak force (all individuals and men) in crude model in study by Rossato et al. (2020) [24], which did not remain significant in the adjusted model. A large cohort study also showed an indirect positive association for higher intake of fatty fish (rich source of omega-3) and higher in grip strength [25]. Most previous studies used single muscle strength outcomes, whereas the current study provides further evidence by studying the association for omega-3 PUFAs in relation to multiple physical function measures. In addition, the systematic review and meta-analysis indicated lack of sufficient data on omega-3 with muscle mass and strength [23]. The study hypothesis was that higher dietary ALA, EPA, DHA and total omega-3 PUFA intake associate with better clinical markers of muscle function and muscle mass and lower fart mass among older women aged 65–72 years.

## Methods

### Study design and participants

Data for the present study were collected from the Osteoporosis Risk Factor and Prevention-Fracture Prevention Study (OSTPRE-FPS), which began in 2003 in Kuopio, Finland [24]. The OSTPRE-FPS was a randomized population-based open trial with a 3-year follow-up in 3,432 women (aged 66–71 years). The primary aim of the study was to determine whether vitamin D and calcium supplementation would be effective in preventing falls and fractures in postmenopausal women. For this sub study to investigate the effect of vitamin D and calcium supplementation on fracture prevention a sample of 750 women (375 from each of the intervention and control groups) was randomly selected from the 3432 women at the baseline underwent detailed examination at baseline and follow-up, including measurements of body composition, clinical, physical function, and laboratory tests. At the end of the trial (*n* = 593, 79.0%), 306 (40.8%) and 287 (38.2%) subjects in the intervention and control groups of the subsample, respectively.

Only at the baseline 554 (93%) women returned a valid food record at the baseline, and 39 women did not return the food record, or it was incomplete. Thus, for this cross-sectional study, the final analytical data included 554 women, with available dietary and physical function variables. The power analysis was performed based on the incidence of fractures [24]. There was no a priori power analysis to calculate the size of the subsample of 750 women randomly selected from the 3432 women at the baseline. All participants provided written permission for participation. The ethical committee of Kuopio University Hospital approved the study in October 2001. The study was registered at Clinical trials.gov by the identification NCT00592917.

### Dietary intake

Dietary intake was assessed using 3-day food records. The records and instructions on completing the diary were sent to the subjects beforehand and they were asked to return the diaries on a research visit at the Kuopio Musculoskeletal Research Unit (KMRU) at the University of Kuopio. Subjects were advised to complete the diary information for 3 consecutive days, including 2 weekdays and either Saturday or Sunday. The types of fats used in bread, in cooking and baking were asked separately. Any unclarities in the diaries were checked by phone calls to the subjects by a nutritionist. Intake of different fatty acids and other nutrients from food records were calculated using the Nutrica program (version 2.5, Finnish Social Insurance Institute, Turku, Finland) [25].

### Body composition measurements

Height and weight of participants were measured in light indoor clothing without shoes, body mass index (BMI) was calculated by weight (kg) divided by height squared (m). Fat mass and lean mass were measured by dual-energy X-ray absorptiometry (DXA) by specially trained nurses. The DXA measurements carried out using the same Lunar Prodigy adhering to the imaging and analysis protocols provided by the manufacturer (Lunar Co., Madison, WI, USA) [26]. DXA is currently a common tool suitable for estimation of body composition in terms of evaluating the ratio between fat, muscle, and bone in different parts of the body [27]. DXA also has been showed to be superior to bioimpedance for estimation of the body composition. Relative skeletal muscle index (RSMI) was calculated as the sum of the non-fat, non-bone skeletal muscle in arms and legs divided by height squared (m^2^).

### Physical performance measurements

Physical performance measures were assessed by trained nurses, including: hand grip strength (kg), number of chair rises in 30 s, ability to squat to ground, knee extension (kg), walking speed 10 m (m/s) and tandem walk for 6 m (m/s), standing with closed eyes for 10 s and one leg stance performance for 30 s; See Isanejad et al. 2016 [28] for details. Hand grip strength was measured in a controlled sitting position with a pneumatic hand-held dynamometer (Martin Vigorimeter, Germany) by calculating the mean of three successive measurements from the dominant hand.

### Short physical performance battery

Short physical performance battery (SPPB) score was calculated using three individual measures of physical performance including walking speed 10 m (m/s), chair rises in 30 s and one leg stance performance categorized in quartiles [6], based on European Working Group on Sarcopenia definition [6] Each quartile was scored on scale of 1–4 points with the total score ranging to 12; higher scores of SPPB indicates better performance.

### Confounders

Confounders were selected based on our prior work and literature reviews on dietary and physical function association [29], including age (years), energy intake (Kcal), protein intake (g/d), dietary vitamin D (µg/d), dietary calcium (mg/d), BMI (kg/m^2^) (or height for body composition analyses), hormone therapy (used, never used and current), smoking (smoker, nonsmokers), osteoporosis (T and Z bone mineral density score for 2SD below the reference population), alcohol consumption (g/week) from a separate questionnaire, physical activity (hours/week) and intervention group. Details of computing the final physical activity variable has been published elsewhere [30]. Among the leisure and exercise variables the most common activities reported were skiing, walking, cycling, swimming, and aerobic exercise, explaining over 90% of the weekly physical activity, which was used to form the physical activity variable. The reported amount of weekly physical activity was used to form a long-term physical activity variable by summing up the average weekly physical activity at the baseline. History of diseases and medications data were collected by self-reported questionnaire at the baseline. Multimorbidity was defined as presence of two or more long-term health conditions, including depression, diabetes mellitus, hypertension, rheumatoid arthritis, coronary heart disease, osteoporosis, cancer, and pulmonary disease.

### Statistical analysis

This study did not have inclusion or exclusion criteria and all women who had data on the PUFA dietary intake were included in this study. All statistical analyses were executed using SPSS software version 26 for Windows (IBM Corp., Armonk, NY). All tests were two-sided and a *P* value of < 0.05 was considered significant. Individual characteristics were analysed using ANOVA test for continuous variables and chi-square for categorical variables and presented for dietary quartiles of omega-3 PUFA and ALA. UNIANOVA controlled for selected confounders was used to account for variation in the dependent variables across the quartiles of omega-3 PUFA and ALA. Further, linear regression analyses calculated the standard coefficient β controlled for selected confounders.

We tested the normality of overlay distribution we used the Kolmogrov and Sahapiro tests in the SPSS, and tests were not significant; and fit the normal curve.

Selected confounders for final analytical models were age, energy intake, protein intake, dietary vitamin D, dietary calcium, BMI (replaced with height for body composition analysis), multimorbidity, hormone therapy, smoking, alcohol consumption, physical activity per week, and osteoporosis. We also run a sensitivity analysis by including income per month as surrogate for socioeconomic status into the model, where results did not materially changed.

## Results

The mean age was 67.8 ± 1.8 (*n* = 554), mean BMI was 27.4 kg/m^2^ and 43% of population had multimorbidity at the baseline. Analysis of baseline demographic and selected variables from self-reported questionnaires did not show significant differences according to quartiles of dietary omega-3 PUFA and ALA (Table 1). Among dietary intake variables higher omega-3 PUFA was associated with lower dietary calcium and higher vitamin D intake (Table 1). Higher ALA intake was associated with lower protein intake (g/day), calcium intake (mg/day) and vitamin D intake (μg/day). Table 2 presents results for the dietary omega-3 intakes, alpha-linolenic acid (g/day) 1.4 (0.7), eicosapentaenoic acid (g/day) 0.12 (0.1), docosahexaenoic acid (g/day) 0.28 (0.3).

**Table 1:** Non-dietary characteristics and intake of energy and energy-adjusted intakes of selected nutrients in the quartiles of total omega-3 PUFA and alpha-linolenic acid

Variables	Quartile of total omega-3 PUFA g/day				*P* value	Quartile of dietary alpha-linolenic acid g/day				*P* value
	1: *n* = 140, mean = 0.90	2: *n* = 139, mean = 1.43	3, *n* = 140, mean = 1.99	4, *n* = 135, mean = 3.15		1: *n* = 141, mean = 0.70	2: *n* = 139, mean = 1.07	3: *n* = 136, mean = 1.51	4:* n* = 138, mean = 2.52	
Age (years)^a^	67.8 ± 1.8	68.0 ± 1.8	68.0 ± 2.0	67.6 ± 1.9	0.283	67.8 ± 1.8	68.0 ± 1.9	67.8 ± 1.8	67.8 ± 2.0	0.723
BMI (kg/m^2^)	28.7 ± 4.5	28.6 ± 4.5	29.7 ± 5.0	28.1 ± 4.9	0.752	28.7 ± 4.3	28.7 ± 4.7	29.2 ± 4.7	28.6 ± 5.2	0.979
Physical activity h/week	1.5 ± 1.2	1.5 ± 1.2	1.3 ± 1.09	1.5 ± 1.2	0.268	4.3 ± 2.9	3.4 ± 3.1	4.0 ± 2.9	4.2 ± 3.2	0.222
Multimorbidity^c^	3.8 ± 2.0	4.0 ± 2.1	3.9 ± 2.3	4.2 ± 3.2	0.825	1.6 ± 1.2	1.4 ± 1.1	1.4 ± 1.1	1.3 ± 1.2	0.405
Years since menopause (years)	18.1 ± 4.9	19.2 ± 4.7	18.4 ± 6.1	18.0 ± 5.9	0.66	18.1 ± 5.4	18.5 ± 4.8	18.4 ± 4.9	18.7 ± 6.6	0.479
Current hormone therapy (years)^b^	22.5	23.7	21.7	20.9	0.66	23.2	20.9	24.5	20.3	0.749
Current smoking (%)	6.6	3.6	5.9	3.0	0.574	5.8	2.9	3.7	6.7	0.343
Income per month (euro)	866 ± 305	863 ± 319	828 ± 303	905 ± 266	0.440	828 ± 264	844 ± 316	860 ± 330	870 ± 288	0.533
Dietary variables										
Energy (kcal/day)	1690 ± 382	1457 ± 325	1482 ± 369	1646 ± 360	0.440	1652 ± 367	1501 ± 370	1514 ± 374	1607 ± 361	0.387
Protein (g/day)	66.9 ± 13.0	69.7 ± 10.8	69.3 ± 11.6	67.5 ± 11.5	0.736	71.6 ± 12.7	68.0 ± 10.6	68.8 ± 11.7	64.9 ± 11.2	0.001
Calcium (mg/day)	1043 ± 320	1050 ± 258	965 ± 282	959 ± 307	0.003	1078 ± 305	1026 ± 270	996 ± 286	917 ± 296	0.001
Vitamin D (μg/day)	6.5 ± 4.5	7.3 ± 3.7	8.4 ± 4.7	8.3 ± 5.1	0.001	8.9 ± 5.4	7.1 ± 4.4	7.5 ± 4.4	7.0 ± 3.9	0.003
Alcohol intake per week (g/day)	8.7 ± 4.5	10.1 ± 4.8	10.4 ± 16.0	10.3 ± 7.8	0.806	10.4 ± 3.6	10.1 ± 4.6	8.8 ± 5.7	10.3 ± 3.1	0.850

^a^ANOVA analysis calculated means and standard deviations for continuous variables

^b^Chi-square tests calculated the n and % for categorical variables

^c^Multimorbidity was defined as presence of two or more long-term health conditions

**Table 2:** Dietary omega 3 PUFAs intake

Dietary omega 3 intake	Means ± SD (*n* = 554)
Alpha-linolenic acid (g/day)	1.4 ± 0.7
Eicosapentaenoic acid (g/day)	0.12 ± 0.1
Docosahexaenoic acid (g/day)	0.28 ± 0.3

Table 3 presents the association of physical function and body composition for omega-3 PUFA and ALA quartiles. After controlling for selected confounders, higher quartile of omega-3 PUFA and ALA were associated with higher SPBB score (*P* = 0.020 and *P* = 0.010, respectively), longer one leg stance (*P* = 0.050 and *P* = 0.035, respectively), faster walking speed 10 m (*P* = 0.048 and *P* = 0.005, respectively), and lower frequency of women with inability to squat to the ground (*P* = 0.014 and *P* = 0.004, respectively). Additionally, higher quartile for ALA was associated with lower fat mass (*P* = 0.001), and higher grip strength (*P* = 0.047).

**Table 3:** Baseline physical function and body composition assessments in the quartiles of total omega-3 PUFA and alpha-linolenic acid ^a^

	Quartile of total dietary omega-3 PUFA g/day				*P* for trend	Quartile of dietary alpha-linolenic acid g/day				*P* for trend
Short physical performance battery	7.7 ± 2.4	8.3 ± 2.3	7.8 ± 2.3	8.8 ± 2.1	0.020	7.7 ± 2.3	7.7 ± 2.2	8.2 ± 2.5	8.8 ± 2.1	0.010
Grip strength (kg)	25.9 ± 4.6	26.1 ± 5.1	25.6 ± 4.5	26.4 ± 4.7	0.672	25.9 ± 4.4	25.8 ± 4.5	25.7 ± 4.9	26.7 ± 4.6	0.047
One leg stance 30 s	18.9 ± 10.1	20.6 ± 9.5	20.1 ± 9.4	22.0 ± 9.2	0.050	18.9 ± 7.1	19.3 ± 7.2	20.1 ± 6.8	23.2 ± 7.4	0.035
Chair rises in 20 s	8.9 ± 7.6	8.3 ± 1.9	7.9 ± 2.3	7.1 ± 2.2	0.223	7.8 ± 5.2	8.3 ± 5.5	7.8 ± 3.0	7.7 ± 2.4	0.666
Tandem walk speed 6 m (m/s)	0.30 ± 0.09	0.37 ± 0.55	0.31 ± 0.09	0.35 ± 0.26	0.371	0.38 ± 0.08	0.31 ± 0.10	0.36 ± 0.53	0.35 ± 0.23	0.680
Walking speed 10 m (m/s)	1.62 ± 0.30	1.69 ± 0.32	1.71 ± 0.30	1.78 ± 0.30	0.048	1.60 ± 0.28	1.62 ± 0.27	1.67 ± 0.31	1.78 ± 0.34	0.005
Standing with eyes closed 10 s (%)	0.96 ± 0.18	1.00 ± 0.20	1.0 ± 0.00	0.96 ± 0.20	0.057	0.97 ± 0.18	0.98 ± 0.13	0.99 ± 0.100	1.0 ± 0.11	0.147
Ability to squat to the ground (%)	10	6	4	3	0.014	11	3	6	4	0.004
Knee extension (kg) ^c^	3.0 ± 0.98	3.1 ± 0.64	3.0 ± 0.83	3.0 ± 0.80	0.813	2.9 ± 0.93	3.0 ± 0.68	3.11 ± 0.82	3.17 ± 0.79	0.245
Lean mass (kg)	39.7 ± 4.4	40.0 ± 3.7	40.0 ± 4.4	40.7 ± 4.3	0.715	39.8 ± 4.0	39.8 ± 4	40.1 ± 4.3	40.1 ± 4.2	0.127
Relative muscle index (kg)	6.75 ± 0.63	6.76 ± 0.59	6.70 ± 0.69	6.80 ± 0.70	0.648	6.7 ± 0.6	6.6 ± 0.6	6.7 ± 0.6	6.7 ± 0.6	0.073
Fat mass (kg)	29.5 ± 8.6	29.2 ± 8.8	28.5 ± 8.0	28.3 ± 9.1	0.812	29.4 ± 7.7	29.1 ± 8.3	27.8 ± 8.1	26.1 ± 7.4	0.001

^a^UNOANOVA adjusted for age, energy intake, protein intake, dietary vitamin D, dietary calcium, BMI, hormone therapy, smoking, osteoporosis, alcohol consumption, physical activity hour/per week and intervention group

Table 4 shows results for the linear regression analysis controlling for selected confounders (age, energy intake, protein intake, dietary vitamin D, dietary calcium, BMI, hormone therapy, smoking, osteoporosis, alcohol consumption, physical activity hour/per week and intervention group). Both omega-3 PUFA and ALA were positively and significantly associated with SPPB (*β* = 0.108, *P* = 0.039 and *β* = 0.118, *P* = 0.034, respectively), one leg stance (*β* = 0.102, *P* = 0.041 and *β* = 0.119, *P* = 0.010, respectively), walking speed (*β* = 0.110, *P* = 0.028 and *β* = 0.113, *P* = 0.047, respectively), and with lower inability to squat to ground (*β* = 0.110, *P* = 0.028 and *β* = 0.112, *P* = 0.027, respectively). Also, ALA intake was positively associated with knee extension force (*β* = 0.075, *P* = 0.037) and inversely associated with fat mass (*β* = − 0.081, *P* = 0.034). Similar associations were observed using 1-SD increment of omega-3 PUFA, ALA, EPA and DHA dietary intake (data not shown).

**Table 4:** Cross-sectional association of dietary omega-3 PUFA and physical function and body composition. PUFA, polyunsaturated fatty acid; add EPA, DHA and ALA

	Total omega-3 PUFA		EPA		DHA		Alpha-linolenic acid	
	Standardized coefficient *β*	*P*^a^	Standardized coefficient *β*	*P*^a^	Standardized coefficient *β*	*P*^a^	Standardized coefficient *β*	*P*^a^
Short physical performance battery	0.108	0.039	0.015	0.752	0.013	0.752	0.118	0.024
Grip strength (kg)	0.018	0.679	0.022	0.983	0.024	0.592	0.013	0.578
One leg stance 30 s	0.102	0.041	0.001	0.730	0.008	0.850	0.119	0.010
Chair rises in 20 s	− 0.043	0.329	− 0.286	0.775	− 0.013	0.487	− 0.014	0.348
Tandem walk speed 6 m (m/s)	0.035	0.451	− 0.017	0.715	− 0.030	0.411	0.058	0.216
Walking speed 10 m (m/s)	0.110	0.028	0.028	0.507	0.026	0.537	0.113	0.047
Standing with eyes closed 10 s (%)	− 0.017	0.696	− 0.039	0.377	− 0.038	0.387	0.003	0.943
Ability to squat to the ground (%)	0.110	0.028	0.025	0.545	0.034	0.410	0.112	0.027
Knee extension (kg) ^c^	0.039	0.394	− 0.096	0.039	− 0.104	0.075	0.096	0.037
Lean mass (kg)	− 0.001	0.976	− 0.029	0.471	0.048	0.158	0.030	0.372
Relative muscle index (kg)	0.009	0.832	0.004	0913	0.039	0.345	0.017	0.684
Fat mass (kg)	0.001	0.970	0.001	0.979	0.013	0.740	− 0.081	0.034

^a^Adjusted for age, energy intake, protein intake, dietary vitamin D, dietary calcium, BMI, hormone therapy, smoking, osteoporosis, alcohol consumption, physical activity hour/per week and intervention group

## Discussion

The main result of the present cross-sectional study was that dietary intake of ALA and total omega-3 PUFA were positively associated with physical function assessments, including faster walking speed 10 m, better performance at one leg stance, ability to squat to the ground and SPPB. Higher ALA quartile was also associated with greater grip strength and lower fat mass. Findings showed no statistically significant association for dietary EPA and DHA with physical function assessments.

Although previous studies reported association between omega-3 PUFA intake with walking speed [31, 32], peak force and knee extension [33]. Robinson et al. found that fatty fish intake was the largest predictor of hand grip strength [34]. Although not directly this suggests that omega-3 PUFA may at least partially explain this association. In a cross-sectional study among French community-dwelling older adults, a higher proportion of the omega-3 PUFAs EPA and DHA in plasma was associated with a higher gait speed [35]. Similar cross-sectional result was reported among 1273 older adults involved in the InCHIANTI study, significant positive association between omega-3 PUFA with the faster completion of a 7-m walking test [36]. Recent study by Rossato et al. [37] showed that the intake of total omega-3 PUFA was positively associated with peak force in older men but not in women. The present study complemented the previous findings in only women, showing where positive association for total omega-3 and ALA was detected with a wider range of physical function outcomes including positive association with one leg stance, ability to squat and grip strength and SPPB. In contrary, the study by Fougere [31] among 400 participants aged 75.2 (± 4.3) years, reported negative association between high levels of baseline red blood cell omega-3 PUFAs and SPPB, the reason for this negative findings was partially explained by the ceiling effect as the mean SPPB was considered particularly high (10.7 out of 12) and a narrow range could reduce that statistical power, whereas in present study SPPB mean was 7.7 out of 12.

Robust assessments indicated in the RCTs for direct effects omega-3 PUFAs on muscle mass and function in the older adult population are scarce. Findings of study by Smith et al. showed that six months omega-3 PUFA therapy in sixty healthy 60- to 85-year-old men and women, increased thigh muscle volume, and handgrip strength, and tended to increase average isokinetic power compared to control group (receiving corn oil) [38]. Another RCT of omega 3 fatty acids supplementation showed no statistically significant differences either in muscle mass or in the hand grip and time up and go tests compared to control group [39]. Identifying the seldom effect of EPA and DHA on muscle mass and strength in the elderly is not feasible based on limited studies, however, a recent meta-analysis of randomized controlled trials (RCTs) found that supplementation with both types of n-3 PUFAs (EPA and/or DHA) elicits a ~ 0.33 kg increase in muscle mass. In addition, EPA and DHA have positive effect on the muscle protein synthesis or reducing oxidative stress in animal models [40, 41].

Currently lower extremity functions are the main indicator for mobility as a clinical screening tool [42], and it maybe that by ageing lower extremity function are more prone to change and perhaps influenced by external stimuli such as diet and exercise. ALA is a plant-derived n-3 fatty acid that mainly exists in flaxseed, soybean, perilla, walnut, and canola oils. In healthy adults, only 5–10% and 2–5% of ALA can be converted into EPA and DHA, respectively [43]. Also, the conversion of ALA to EPA and DHA in men is estimated to be as low as < 8% and < 4% respectively, whilst in women it is slightly higher at 21 and 9%, respectively [44]. Therefore, inter-individual variability should be considered in future studies and possible EPA and DHA supplementation.

Our study population is characterized by a high intake of omega-3 PUFA and ALA. The analysis of 3-day food record in this study showed that OSTPRE-FPS older women consumed EPA + DHA 0.41 ± 0.47 (g/day), ALA 1.4 ± 0.8 g/day (corresponding to 0.83% of the mean energy intake), and total omega-3 PUFA 8.8 ± 3.4 g/day, which all are within the range of recommendation by Nordic Nutrition recommendation 2012: minimum 1 E% from n-3 fatty acids and intake of EPA + DHA up to 200–250 mg/day.

There are multiple potential mechanisms which can explain the observed association in this study. Low grade inflammation has been considered as important trigger for inflammageing occurring with aging, which is a chronic state of slightly increased plasma levels of pro-inflammatory mediators, such as tumor necrosis factor α (TNF-α), interleukin 6 (IL-6) and C-reactive protein (CRP) [45]. Although the causative link between inflammatory markers and the risk for functional decline and mortality in elderly are hard to established, strong correlation between these two have been identified in previous studies [46]. Without pronouncing the causal links between inflammatory mediator and physical function, the current literature suggests that nutritional factors and particularly omega-3 PUFA are among the biological mechanism to reduce low grade inflammation which can play role to delay or prevent the onset of physical function decline [16, 47]. Results from a study indicated that ALA decreases the plasma level of inflammatory cytokines (TNF-alpha and IL-6) in the elderly [40]. In addition, findings from experimental studies, although limited, suggest that omega-3 PUFA contribute to muscle protein synthesis anabolic pathway. This finding was consistent with the animal models and cell culture suggesting that omega-3 PUFA can enhance the muscle protein synthesis [48]. Current evidence also suggest that omega-3 PUFAs are potentially able to enhance the capacity of muscle cells for more permeable reaction with regard to necessary nutrients, such as glucose and amino acids [8]. The finding of this study on positive association for omega-3 PUFA, and ALA with physical function outcomes can be explained by these underlying mechanisms, however, further studies on effect of omega-3 PUFAon muscle function and muscle protein synthesis are required. Besides, the positive finding may be explained by the indirect relationship of omega-3 PUFA as they can improve blood lipid profile and heart function [49] which in turn are associated with healthy ageing [40, 50]. Previously, we have published that Mediterranean and Baltic Sea diets have positive associations with improved physical function and lean mass in this data [29]. We acknowledge that only for ALA the correlation coefficients relating to Mediterranean diet (*r* = 0.210, *P* < 0.001) and Baltic sea diet were statistically significant (*r* = 0.126, *P* = 0.004), but not with EPA or DHA. Even though we cannot exclude other confounding factors it maybe suggesting that role of fatty acids should be considered more in the whole dietary approach rather than single nutrients which may explain the inconsistent results of previous studies.

There are possible limitations to consider. We conducted secondary analysis on data from a RCT and the RCT was not originally designed to investigate the associations reported here from the baseline of the study. Our study population included only relatively healthy older women from a homogenous Finnish population, thus results may not be generalized to other older populations. We controlled for multiple confounders; the probability of other lifestyle factors related to higher omega-3 PUFA intake affecting physical functions cannot be excluded. Finally, since the nature of this study is observational and using self-assessment tools such as food diary as the data source, it might be that dietary intake including omega-3 PUFA can be over or underestimated.

## Conclusion

In conclusion, this study in older women suggests that higher omega-3 PUFA and ALA s were positively associated with measures of physical capacity (walking speed 10 m, at one leg stance, ability to squat to the ground and SPPB and grip strength) but not with the muscle mass. However, there is considerable scope for future work to fully elucidate the potential of omega-3 PUFA in muscle function as well as on possible gender differences of omega-3 function in men and women.
